# Association between Chlamydial Infection with Ectopic and Full-Term Pregnancies: A Case-Control Study

**DOI:** 10.3390/tropicalmed7100285

**Published:** 2022-10-06

**Authors:** Valliammai Jayanthi Thirunavuk Arasoo, Mariyammah Masalamani, Amutha Ramadas, Nisha Angela Dominic, Darien Daojuin Liew, Robin Wai Jen Sia, Anuradha Wanigaratne, Keshawa Weerawarna, William Lik Loong Wong, Ravichandran Jeganathan

**Affiliations:** 1 Jeffrey Cheah School of Medicine and Health Sciences, Monash University Malaysia, Bandar Sunway 47500, Selangor, Malaysia; 2 Department of Obstetrics and Gynaecology, Hospital Sultanah Aminah Johor Bahru, Johor Bahru 81200, Johor, Malaysia

**Keywords:** ectopic pregnancy, chlamydia infection

## Abstract

Ectopic pregnancies (EPs) are potentially fatal if not recognized early. Evidence of an association with chlamydial infection in South East Asia is lacking. This case-control study aims to (i) compare chlamydial infection in women with EP to women who delivered a full-term pregnancy, (ii) investigate classical factors associated with EP, and (iii) investigate rupture status in EP. Seventy-two women with a confirmed diagnosis of EP and sixty-nine who delivered a full-term pregnancy in a tertiary hospital in Malaysia were recruited from November 2019 to January 2022. Demographic and relevant clinical data and intraoperative findings were documented. Blood samples for testing IgG levels of chlamydia were obtained. Women with EP were more likely to have tested positive for chlamydia than those with a full-term delivery (34.7% vs. 13.0%, AOR = 4.18, 95% CI = 1.67–10.48, *p* = 0.002). The majority did not have the classic risk factors associated with EP. An amount of 52.8% presented with a ruptured EP, with 84.2% of ruptures occurring after six weeks of gestation. An amount of 44.2% had an estimated blood loss of more than 500 cc, with 20% losing more than 1500 cc of blood. The prevalence of prior chlamydial infection in women with EP is significant enough to necessitate a review of early pregnancy care.

## 1. Introduction

Ectopic pregnancy (EP) was the fourth leading cause of maternal mortality in Malaysia in the years 2019 and 2020 [1]. Common risk factors include infections, surgery, smoking, in vitro fertilization, previous EP, previous pelvic surgery, and intrauterine device use [2,3,4]. Although the exact pathogenesis of EP is unknown, it is hypothesized that impaired embryo tubal transportation and alterations in the tubal environment result in embryo retention within the fallopian tubes [2,3]. 

The tubal environment is governed by sex hormones, cytokines, growth factors, expression proteins, and the surveillance of immune cells. Infection-stimulated cytokines are involved in the development of EP. There is an increased interleukin-6 (IL-6) level and leukemia inhibitory factor (LIF) in the fallopian tubes of women with EP [3]. Epithelial shedding due to chronic salpingitis is thought to result in the exposure of the stromal surface in the fallopian tube, producing high levels of the LIF, facilitating the implantation of the arrested embryo [2]. Prior studies postulate that EP could be linked to chronic pelvic inflammation [2,5,6].

*Chlamydia trachomatis* (*C. trachomatis*) has been reported as the most common pathogen that leads to sexually transmitted infections (STIs) [3,7]. The World Health Organization estimates that in 2016, there were 376 million new infections, with one in four being STIs: chlamydia (127 million), gonorrhea (87 million), syphilis (6.3 million), and trichomoniasis (156 million). *C. trachomatis* is associated with pelvic inflammatory disease, infertility, and EP [8]. Other common bacterial infections include *Neisseria gonorrhoea* and *Mycoplasma genitalium* [3]. In healthy women, the expression of intrauterine integrins is associated with successful intrauterine implantation. However, a prior *C. trachomatis* infection results in increased integrin expression in the tubal epithelial cells, which likely promotes trophoblast attachment leading to EP [9]. 

A recent meta-analysis concluded that EP was more likely in pregnant women who tested positive than those who did not [6]. Although this meta-analysis categorized eight studies as Asian, only two studies were from South East Asia, namely, Thailand [10] and Vietnam [11], which reported relatively smaller sample sizes. Additionally, studies exploring the prevalence of chlamydia infection in Malaysia were targeted at specific populations such as the urban population [12], prostitutes [13], and those seen at the infertility clinic [14,15]. As there is a scarcity of regional data on the prevalence of *C. trachomatis* infection, presenting features, and intraoperative findings in patients with EP, this study could provide some insight. 

Hence, we aimed (i) to compare the prevalence of prior chlamydial infection in women with EP and women who delivered from 37 weeks onwards, (ii) to assess the contribution of chlamydial infection and associated risk factors to EP, and (iii) to document the period of gestation when an EP is more likely to rupture. 

## 2. Materials and Methods

### 2.1. Study Design

This was an unmatched case-control study conducted in Hospital Sultanah Aminah Johor Bahru, Malaysia, among adult women (>18 years) with a confirmed diagnosis of EP (case) or admitted for delivery from 37 weeks onwards (control). We obtained ethical approval from the Malaysian Research Ethics Committee (MREC) (NMMR: 18-3214-44797) and the Monash University Human Research Ethics Committee (MUHREC) (ID: 18558) prior to the commencement of the study.

### 2.2. Recruitment and Data Collection

Consenting patients aged 18 and above admitted for management of a confirmed diagnosis of EP were recruited with informed consent. Patients who were clinically stable either pre- or post-operative were recruited. Controls were those aged 18 and above admitted for delivery from 37 weeks onwards. Only those in the latent phase of labor or admitted for labor induction were recruited as controls. Patients above 18 who had impairments such as hearing impairment were excluded.

Demographic data that were collected included age, ethnicity, marital status and education level. Relevant clinical data recorded included the number of sexual partners, parity status, risk factors for EP, and clinical findings. These are tabulated in Table 1 and Table 2. Blood samples totaling 5 mL were obtained for testing IgG levels of chlamydia. Detection of chlamydia IgG was performed by an independent external lab using CHLG-chlamydia IgG (immunoblot), and a result, >1.00 was considered positive.

The study recruitment and primary data collection took place at Hospital Sultanah Aminah Johor Bahru, Malaysia, between November 2019 and January 2022. Although an average of two patients with EP were treated per week, data collection was paused during the COVID-19 pandemic due to the government’s implementation of the movement control order (MCO). At this center, the primary method of treatment employed for EP was the surgical approach (either laparoscopic or laparotomy). Patients who were clinically stable underwent the laparoscopic approach. However, if there were significant challenges intraoperatively or if the patient had significant hemoperitoneum, the approach was converted to laparotomy.

### 2.3. Sample Size Justification

The minimal sample size was estimated using OpenEPI [16] based on the difference in the proportion of outcome (EP) according to parity (1 vs. >3) as reported by Mpiima et al. [17] The authors reported the prevalence of ectopic pregnancy among those in parity of 1 (unexposed) to be 2.38% as opposed to those with parity >3 (exposed) which was 18.75%. A minimum sample size of 66 was required in each group to give the study 80% power at α = 0.05 (two-sided).

### 2.4. Statistical Analysis

Data collected were tabulated and analyzed using IBM^®^ SPSS^®^ Statistics Ver.26.0. Descriptive statistics such as frequencies, percentages, means, and standard deviations were used to describe the study participants’ characteristics. Associations between categorical variables and study groups were determined with Pearson’s chi-square or Fisher’s exact tests. Mean values of continuous variables were compared between the study groups using independent t-tests. Multivariable logistic regression (enter method) was used to determine the relationship between chlamydia infection and EP. Variables with a *p* value >0.25 in the bivariate analysis were adjusted for in the multivariate model. The model’s goodness-of-fit was determined using the Hosmer–Lemeshow test. The adjusted model was also assessed for multicollinearity and interactions between variables. Statistical significance was set at *p* < 0.05.

## 3. Results

Seventy-two patients with EP and sixty-nine women with a non-ectopic full-term pregnancy between 37 and 40 weeks of gestation were recruited for the study. The mean age of the study patients was 30.16 years (SD = 5.07 years). The majority of them were of Malay ethnicity (79.1%), were Muslim (80.1%), had at least a secondary level of education (97.2%), and were employed (66%). More than 80% of the patients and their partners were married for the first time and had only one partner. None of the demographic characteristics differed between the study groups (Table 1). 

Nine study patients had pelvic surgery performed within the past year. Three patients underwent lower segment caesarean section (LSCS); others underwent one of the following surgeries: appendectomy, dilatation and curettage (D and C), endometrial polypectomy laparoscopic myomectomy, laparoscopic salpingectomy, and salpingectomy. Although only eight patients had a smoking history, those with EP had been smoking for significantly longer than controls (14.60 ± 6.02 years vs. 2.97 ± 4.36 years, *p* = 0.028). The remaining clinical characteristics did not differ between study groups (Table 2).

Almost a quarter of all pregnancies were also infected with chlamydia, as observed with the presence of chlamydia IgG antibodies (Table 3). Chlamydia infection was more prevalent in EP patients compared to non-ectopic pregnancy patients (34.7% vs. 13.0%). After adjusting for potential confounding factors, the odds for chlamydia infection were higher than four-fold in EP patients than in the control group (aOR = 4.18, 95% CI = 1.67–10.48, *p* = 0.002).

In subsequent analysis, we explored the association between EP-related characteristics and chlamydia IgG antibodies (Table 4). Per vaginal bleeding was twice more common in chlamydia-infected EP cases compared to EP cases negative for the infection (50.0% vs. 24.0%, *p* = 0.033). The choice of management options also differed between those with and without infection (*p* = 0.002). Laparotomy salpingectomy was the most common management choice among all patients (47.9%), especially those infected with chlamydia (60.0%). However, laparoscopic salpingectomy was more common among those without the infection (56.5%). Other variables were not found to be associated with chlamydia infection.

No association between EP rupture status and gestation period was found (Table 5).

## 4. Discussion

In our study, 34.7% of women with EP and 13.0% who delivered full term were IgG positive for chlamydia. Very few studies in South East Asia report the prevalence of chlamydia infection in women with EP. An analysis of 177 women with EP in Vietnam reported that only 24.9% tested IgG positive for chlamydia [11]. In a small study of 32 Thai women with EP, although 34.38% had chlamydia DNA in the fallopian tube, only 21.88% had serum-specific IgG for chlamydia. However, no strong independent association between EP risk and chlamydia antibodies was demonstrated [10]. Detecting chlamydial antibodies decreases with time since the initial infection. However, a higher proportion of women with more than one diagnosed chlamydial infection have detectable antibody levels beyond six months [18]. Based on these two studies [10,18], it can be postulated that our sample of women with EP had chlamydial infections much earlier and tested negative for the antibodies. It could also suggest that the women in our study who tested positive were possibly in the first six months of initial infection or had more than one episode of infection. 

Although we reported a higher prevalence of chlamydia antibodies in our participants compared to others from the region, a study conducted in Uganda reported double our figures, where 60% of women with EP and 26.32% of those with an intrauterine pregnancy had chlamydia antibodies [17]. This may be attributed to differences in the prevalence of chlamydia in the general population. Malaysia currently does not have data on the prevalence of chlamydia infection in the general population.

Intraoperatively, only 16.0% of those who were IgG positive and 4.4% who were negative had peri tubal adhesions on at least one side. A developed mathematical model estimated that 10% of untreated chlamydial infections could result in PID [19]. Our findings were not that far off from the estimate of the mathematical model. Peri tubal adhesions in our study were not classified according to any validated scoring system. It should be noted and included in patient counselling that a higher revised American Fertility Society score is associated with a higher occurrence of repeat EPs [20]. 

Traditional risk factors associated with EP include a history of PID, smoking, history of pelvic surgery, and use of copper IUCD. In our study, none of these factors were significantly associated with EP. Similar findings were reported by another study [21]. Hence, a meta-analysis should be conducted to understand the usefulness of traditional risk factors in current times.

Although there were twenty-five women with EP and nine women with a full-term pregnancy who tested positive, they largely did not report symptoms to suggest PID. This finding is not unique to our study, as other studies have also reported that chlamydia seropositivity was more common than a documented or reported history of infection [22,23]. 

Other microorganisms such as *Mycoplasma genitalium* [24] and *Neisseria gonorrhoea* [25] also cause PID that can damage the tubes resulting in EP. Although we did not test for these microorganisms, it is known that *C. trachomatis* infection occurs more frequently than *M. genitalium* or *N. gonorrhoea* infection.

A prospective cohort study of 5704 Dutch women (29.5% chlamydia positive) reported an EP incidence rate of 0.8 per 1000 person-years (0.4 to 1.5) in those who had prior chlamydia infection and 0.6 per 1000 person-years (0.4 to 0.6) for women who were chlamydia negative [26]. The incidence of EP in both groups was approximately the same. This, combined with our findings that 65.5% of women with EP tested negative for chlamydia and had no association with traditional risk factors, leads to the question of whether another factor facilitates blastocyst implantation in the fallopian tube before it reaches the uterine cavity. There is also the possibility of undetected microbes colonizing the tubes or even dysbiosis.

Our study aims included documenting the period of gestation (POG) that tubal EP ruptured. Ruptures were seen in 38 (52.8%) women with EP. Although there was no statistically significant specific POG that rupture occurred in our study, 84.2% of EPs ruptured after a six-week POG. Patient factors that led to a delay in seeking early pregnancy care were not explored in our study. This suggests that early self-tests and seeking pregnancy care earlier should be encouraged. A study of 48 women with ruptured EP and 51 women with unruptured EP, reported that the POG for a ruptured EP was 8.0 ± 0.9 weeks and 7.3 ± 1.0 weeks for an unruptured EP. However, these values were statistically insignificant [27]. In another study of 144 women with ruptured EP and 79 women with unruptured EP, a rupture occurred at 53.9 ± 4.7 days (7.7 ± 0.7 weeks). This finding was reported as borderline significant [21]. The plausible reason we failed to document any significant POG for tubal rupture is that the POGs recorded in our study were largely calculated based on the self-reported first day of the last menstrual period, thus, making them less objective.

Approximately 44% (*n* = 31) of the EPs had significant blood loss of more than 500 mls, with approximately half of them having a massive hemorrhage, likely requiring blood transfusion. Approximately 45% (*n* = 14) of this group were IgG positive for chlamydia, while the remainder were negative, which was not statistically significant. However, this degree of hemorrhage remains a contributing factor to maternal morbidity and mortality, as reflected by our recent statistics [1].

Our study is one of the very few studies from South East Asia that documented the presence of chlamydial infection in women with EP compared to women who delivered full term. We found an alarmingly high presence of prior chlamydial infection in women with EP and an unexpected presence of prior infection in women who delivered full term.

A limitation of this study is that we tested only for chlamydial infection and not for other microorganisms such as *M. genitalium* and *N. gonorrhoea*. Another limitation is that the test kit did not discern the type of chlamydia infection. However, *Chlamydia psitacci* infection is uncommon in our population.

## 5. Conclusions

EP continues to be a significant cause of maternal mortality in Malaysia. Early diagnosis and prompt management are crucial as ruptures tend to occur after a six-week POG. Although chlamydial infection is significantly associated with EP, almost two-thirds of the women with EP in our study did not have the infection. Relying on traditional textbook risk factors to formulate a diagnosis is not helpful as our study reported no significant risk factors. A meta-analysis could be conducted to understand the usefulness of traditional risk factors in current times. The authors believe that further research should explore the role of female reproductive tract dysbiosis and the role of passive smoking as causative factors of EP. Research into the duration from which infection occurs until the time EP results could explain why some women with prior chlamydial infection delivered a full-term pregnancy.

## Figures and Tables

**Table 1:** Demographic characteristics of the study patients (N = 141).

		All Patients	Ectopic Pregnancy (Case)	Non-Ectopic Full-Term Pregnancy (Control)	*p* Value
		(N = 141)	(*n* = 72)	(*n* = 69)	
Age (years)	Mean (SD)	30.16 (5.07)	30.44 (4.68)	29.87 (5.47)	0.503
Ethnicity	Malay	112 (79.2)	59 (81.9)	53 (76.8)	0.718
	Chinese	10 (7.1)	4 (5.6)	6 (8.7)	
	Indian	18 (12.8)	9 (12.5)	9 (13.0)	
	East Malaysian Native	1 (0.7)	0 (0.0)	1 (1.4)	
Education	Primary	4 (2.8)	2 (2.8)	2 (2.9)	0.512
	Secondary	72 (51.1)	40 (55.6)	32 (46.4)	
	Tertiary	65 (46.1)	30 (41.7)	35 (50.7)	
Employment	Yes	93 (66.0)	49 (68.1)	44 (63.8)	0.591
	No	48 (34.0)	23 (31.9)	25 (36.2)	
Patient’s marital status	Single	4 (2.8)	2 (2.8)	2 (2.9)	
	First marriage	118 (83.7)	60 (83.3)	58 (84.1)	
	Second marriage or more	19 (13.5)	10 (13.9)	9 (13.0)	
Partner’s marital status	Single	5 (3.5)	2 (2.8)	3 (4.3)	0.816
	First marriage	118 (83.7)	60 (83.3)	58 (84.1)	
	Second marriage or more	18 (12.8)	10 (13.9)	8 (11.6)	
Number of partners	0	1 (0.7)	1 (1.4)	0 (0.0)	0.155
	1	114 (82.0)	60 (85.7)	54 (78.3)	
	2	23 (16.5)	8 (11.4)	15 (21.7)	
	3	1 (0.7)	1 (1.4)	0 (0.0)	

**Table 2:** Clinical characteristics of the study patients (N = 141).

		All Patients	Ectopic Pregnancy (Case)	Non-Ectopic Full-Term Pregnancy (Control)	*p* Value
		(N = 141)	(*n* = 72)	(*n* = 69)	
Gravida	1	49 (34.8)	30 (41.7)	19 (27.5)	0.193
	2–3	58 (41.1)	25 (34.7)	33 (47.8)	
	4–5	23 (16.3)	13 (18.1)	10 (14.5)	
	More than 5	11 (7.8)	4 (5.6)	7 (10.1)	
Parity	0	58 (41.1)	36 (50.0)	22 (31.9)	0.088
	1–2	64 (45.4)	28 (38.9)	36 (52.2)	
	3–4	17 (12.1)	8 (11.1)	9 (13.0)	
	More than 4	2 (1.4)	0 (0.0)	2 (2.9)	
History of EP	Yes	5 (3.5)	4 (5.6)	1 (1.4)	0.367
	No	136 (96.5)	68 (94.4)	68 (98.6)	
History of PID	Yes	5 (3.5)	4 (5.6)	1 (1.4)	0.367
	No	136 (96.5)	68 (94.4)	68 (98.6)	
History of pelvic surgery	Yes	9 (6.4)	4 (5.8)	5 (6.9)	1.000
	No	132 (93.6)	65 (94.2)	67 (93.1)	
History of smoking	Yes	8 (5.7)	5 (6.9)	3 (4.3)	0.719
	No	133 (94.3)	67 (93.1)	66 (95.7)	
Duration of smoking (years)	Mean (SD)	10.23 (7.90)	14.60 (6.02)	2.97 (4.36)	0.028 *
Prior use of copper IUD	Yes	3 (2.1)	1 (1.4)	2 (2.9)	0.614
	No	138 (97.9)	71 (98.6)	67 (97.1)	
Purulent per vaginal discharge	Yes	13 (9.2)	9 (12.5)	4 (5.8)	0.169
	No	128 (90.8)	63 (87.5)	65 (94.2)	
Abdominal pain with fever	Yes	6 (4.3)	4 (5.6)	2 (2.9)	0.681
	No	135 (95.7)	68 (94.4)	67 (97.1)	
Pelvic pain with fever	Yes	1 (0.7)	1 (1.4)	0 (0.0)	1.000
	No	140 (99.3)	71 (98.6)	69 (100.0)	

EP = ectopic pregnancy, PID = pelvic inflammatory disease. * Significant at *p* < 0.05.

**Table 3:** Association between chlamydia IgG antibodies and ectopic pregnancies (N = 141).

		All Patients	Ectopic Pregnancy (Case)	Non-Ectopic Full-Term Pregnancy (Control)
		(N = 141)	(*n* = 72)	(*n* = 69)
Chlamydia IgG antibodies	Positive	34 (24.1)	25 (34.7)	9 (13.0)
	Negative	107 (75.9)	47 (65.3)	60 (87.0)
OR ^a^ (95% CI)			3.55 (1.51–8.31)	1.000
* p * value			0.004 *	
aOR ^b^ (95% CI)			4.18 (1.67–10.48)	1.000
* p * value			0.002 *	

^a^ Unadjusted logistic regression model; ^b^ Adjusted for number of partners, gravidity, and purulent per vaginal discharge. Model fulfilled Hosmer–Lemeshow test (*p* = 0.211), multicollinearity, and interaction assumptions. * Significant at *p* < 0.05.

**Table 4:** Association between ectopic pregnancy-related characteristics and chlamydia IgG antibodies (*n* = 72).

		All EP Patients	Chlamydia IgG Antibodies		*p* Value
			Positive	Negative	
		(N = 72)	(*n* = 25)	(*n* = 47)	
History of EP	Yes	4 (5.6)	1 (4.0)	3 (6.4)	1.000
	No	68 (94.4)	24 (96.0)	44 (93.6)	
History of PID	Yes	4 (5.6)	1 (4.0)	3 (6.4)	1.000
	No	68 (94.4)	24 (96.0)	44 (93.6)	
History of pelvic surgery	Yes	5 (6.9)	0 (0.0)	5 (10.6)	0.156
	No	67 (93.1)	25 (100.0)	42 (89.4)	
History of smoking	Yes	5 (6.9)	1 (4.0)	4 (8.5)	0.652
	No	67 (93.1)	24 (96.0)	43 (91.5)	
Number of partners	0	1 (2.9)	0 (0.0)	1 (0.7)	0.071
	1	25 (73.5)	91 (85.0)	116 (82.3)	
	2	7 (20.6)	16 (15.0)	23 (16.3)	
	3	1 (2.9)	0 (0.0)	1 (0.7)	
Prior use of copper IUD	Yes	1 (98.6)	0 (0.0)	1 (2.1)	1.000
	No	71 (1.4)	25 (100.0)	46 (97.9)	
Period of gestation (weeks)	Unsure/<5	6 (8.5)	2 98.0)	4 (8.7)	0.714
	5–5+6	4 (5.6)	0 (0.0)	4 (8.7)	
	6–6+6	19 (26.8)	9 (36.0)	10 (21.7)	
	7–7+6	11 (15.5)	3 (12.0)	8 (17.4)	
	8–8+6	23 (32.4)	7 (28.0)	16 (34.8)	
	≥9	8 (11.3)	4 (16.0)	4 (8.7)	
Purulent per vaginal discharge	Yes	9 (12.5)	4 (16.0)	5 (10.6)	0.710
	No	63 (84.0)	21 (84.0)	42 (89.4)	
Abdominal pain with fever	Yes	4 (5.6)	1 (4.0)	3 (6.4)	1.000
	No	68 (94.4)	24 (96.0)	44 (93.6)	
Pelvic pain with fever	Yes	1 (1.4)	1 (4.0)	0 (0.0)	0.347
	No	71 (98.6)	24 (96.0)	47 (100.0)	
Ectopic status	Not ruptured	34 (47.2)	9 (36.0)	25 (53.2)	0.164
	Ruptured	38 (52.8)	16 (64.0)	22 (46.8)	
Per vaginal bleeding ^a^	Yes	42 (59.2)	23 (50.0)	6 (24.0)	0.033 *
	No	29 (40.8)	23 (50.0)	19 (76.0)	
Abdominal pain ^a^	Yes	60 (84.5)	20 (80.0)	40 (87.0)	0.501
	No	11 (15.5)	5 (20.0)	6 (13.0)	
Management options ^a^	Laparoscopy salpingostomy	2 (2.8)	1 (4.0)	1 (2.2)	0.002 *
	Laparoscopy salpingectomy	31 (43.7)	5 (20.0)	26 (56.5)	
	Laparotomy salpingostomy	2 (2.8)	2 (8.0)	0 (0.0)	
	Laparotomy salpingectomy	34 (47.9)	15 (60.0)	19 (41.3)	
	Laparotomy cornual resection	2 (2.8)	2 (8.0)	0 (0.0)	
Uterus ^b^	Uniformly enlarged	23 (32.9)	5 (20.0)	18 (40.0)	0.106
	Fibroids	1 (1.4)	1 (4.0)	0 (0.0)	
	Normal	46 (65.7)	19 (76.0)	27 (60.0)	
Peritubal adhesions at least one side ^b^	Yes	6 (8.6)	4 (16.0)	2 (4.4)	0.177
	No	64 (91.4)	21 (84.0)	43 (95.6)	
Peritubal adhesions at both sides ^b^	Yes	2 (2.9)	2 (8.0)	0 (0.0)	0.124
	No	68 (97.1)	23 (92.0)	45 (100.0)	
Blood loss (ml) ^b^	<500	39 (55.7)	11 (44.0)	28 (62.2	0.457
	500–999	12 (17.1)	5 (20.0)	7 (15.6)	
	1000–1499	5 (7.1)	2 (8.0)	3 (6.7)	
	≥1500	14 (20.0)	7 (28.0)	7 (15.6)	

^a^*n* = 71; ^b^ *n* = 70. * Significant at *p* < 0.05.

**Table 5:** Association between gestation period (weeks) and rupture status in ectopic pregnancies.

Period of Gestation (weeks)	All EP Patients	Rupture Status		*p* Value
		Ruptured	Not Ruptured	
	(N = 71)	(*n* = 38)	(*n* = 33)	
<5/unsure	6 (8.5)	4 (10.5)	2 (6.1)	0.517
5–5+6	4 (5.6)	2 (5.3)	2 (6.1)	
6–6+6	19 (26.8)	13 (34.2)	6 (18.2)	
7–7+6	11 (15.5)	4 (10.5)	7 (21.2)	
8–8+6	23 (32.4)	12 (31.6)	11 (33.3)	
≥9	8 (11.3)	3 (7.9)	5 (15.2)	

## Data Availability

The data presented in this study are available on request from the corresponding author. The data are not publicly available due to informed consent conditions.
